# The level of epidermal growth factor receptors expression is correlated with the advancement of colorectal adenoma: validation of a surface biomarker

**DOI:** 10.18632/oncotarget.14961

**Published:** 2017-02-01

**Authors:** Nicolas Williet, Carmen Adina Petcu, Leslie Rinaldi, Michèle Cottier, Emilie Del Tedesco, Léa Clavel, Olivier Dumas, Camille Jarlot, Nadia Bouarioua, Xavier Roblin, Michel Peoc’h, Jean-Marc Phelip

**Affiliations:** ^1^ Department of Hepato-Gastroenterology, University Hospital of Saint-Etienne, France; ^2^ Department of Pathology, University Hospital of Saint-Etienne, France; ^3^ Inserm U1059, Saint-Etienne, France; ^4^ Laboratory of Cytopathology, University Hospital of Saint-Etienne, France

**Keywords:** biomarker, epidermal growth factor, colorectal adenoma

## Abstract

**Introduction:**

Data about the expression of Epidermal Growth Factor Receptors (EGFRs) in colorectal adenomas remain scarce.

**Results:**

101 patients were enrolled including 53 controls. All adenomas (*n* = 38) and CRC (*n* = 5) were EGFR positive. Hyperplastic polyps (HP) (*n* = 8) and control colons (*n* = 53) were EGFR negative in half of cases (*p* < 0.0001). A well significant gradient of increased EGFR expression was observed between adjacent mucosa, hyperplastic lesions, low grade dysplasia (LGD) (*n* = 30), high grade dysplasia (HGD) adenomas (*n* = 9) and cancers (*p* < 0.0001). EGFR overexpression was reported in 100% of cancers, 77.8% of HGD, and 10% of LGD adenomas. By multivariate analysis in adenomas, associated factors with EGFR overexpression were HGD and tubulo-villous feature.

**Materials and Methods:**

All patients undergoing colonoscopy in the university center of Saint-Etienne were eligible to the study from December 2015 to March 2016. In patients with colorectal neoplasia (lesions group), biopsies were performed on the lesion before its resection, and on the adjacent and distal colon mucosa. In control group, biopsies were performed in the right and left side colon. The EGFR expression was assessed by immunohistochemical scores (Goldstein grade, intensity of staining, composite score), using a primary mouse monoclonal antibody (EGFR, clone 113, Novocastra). Outcomes were compared using Kruskal-Wallis and/or Mann-Whitney-U tests, appropriately. The associated clinical, endoscopic and histological factors with EGFR overexpression (composite score ≥ 6) were assessed for adenomas by logistic regression.

**Conclusions:**

EGFR are early involved in colorectal carcinogenesis, and their expression is strongly correlated to the neoplasia stage, leading to validate EGFR as an interesting surface biomarker of adenomas.

## INTRODUCTION

Colorectal cancer (CRC) remains the second most worldwide cause of cancer related death, with 1.3 million new cases diagnosed annually [1]. Screening for CRC has been proven to be effective in reducing CRC incidence and mortality. Colorectal adenoma consists of cells of the mucosa that acquired successively abnormal modifications of their architecture and morphology, from low grade dysplasia (LGD) to carcinoma cells.

Colonoscopy is the gold standard examination tool for the colon and rectum. It is capable of both detection and removal of neoplasia during a single examination. Despite performances of this exam, the risk of interval CRC (cancer occurring between two colonoscopies) is about 0.5% in moderate risk population [2]. This limit is mainly due to a substantial missed lesions rate (20%–26%) [2–4], particularly for small and flat or serrated adenomas in right-side colon (up to 55% in genetically predisposed patients [3]). Despite, improved adenomas detection by the use of dye-based or electronic chromoendoscopy in high risk population, this rate remain significant [3, 5]. Moreover, colorectal carcinogenesis is accelerated in both patients with Lynch syndrome and those with IBD in who there is also some difficulty to distinguish authentical adenomatous lesions from pseudopolyps related to chronic inflammation of the disease.

Nanotechnology is a growing field of science that involves on particles developed at nanoscale (ranging from 1 nm to 100 nm). These nanoparticles consist of nanomolecules which can belabeled with organic dyes, detectable by device able to capture near-infrared (NIR) light spectrum. A part of the nanotechnology Research is focused on the Medical Science, including molecular imaging, such as tomodensitometry and Resonance magnetic imaging. The use of nanoparticles as diagnostic tool during colonoscopy is an emerging approach [6–8]. With selective optical agents functioning in the NIR light spectrum, contrast between normal mucosa and dysplastic tissue could potentially be greatly enhanced, thereby reducing adenomas miss-rates [9]. These agents should target biomarkers known to be overexpressed in colorectal cancer such as epidermal growth factor receptor (EGFR) [10, 11].

However, data remain scares about the level of EGFR expression on colorectal adenomas [12, 13], in contrast with CRC [10–12, 14–18]. The level of EGFR expression varies significantly in CRC, due to the use of variable immunohistochemistry methods across studies. Preclinical studies have suggested early involvement of EGFR in colorectal carcinogenesis [19–21]. In other hand, other studies reported expression of EGFR in normal colonic mucosa. Before to develop diagnostic nanoparticles bound to cetuximab (anti-EGFR antibody currently used in CRC), the level of EGFR expression on adenomas surface should be assessed and compared to normal colorectal mucosa or to other non adenomatous lesions.

Primary objective of our study is to evaluate the level of EGFR expression on colorectal adenomas surface compared to normal cells and CRC.

Secondary objective is to determine clinical, endoscopic or histological factors that could be associated with an overexpression of this receptor.

## RESULTS

### Population study

Of the 304 eligible patients who had colonoscopy from December 2015 to March 2016 in the University Hospital of Saint-Etienne (France), 108 were enrolled. Four patients refused the inclusion. One patient had a right sided colectomy history that was unknown to the endoscopist before the exam. Two patients were excluded because of loss of right side colon sample. Four patients had several colorectal lesions that were separately included for analysis.

**Figure 1:**
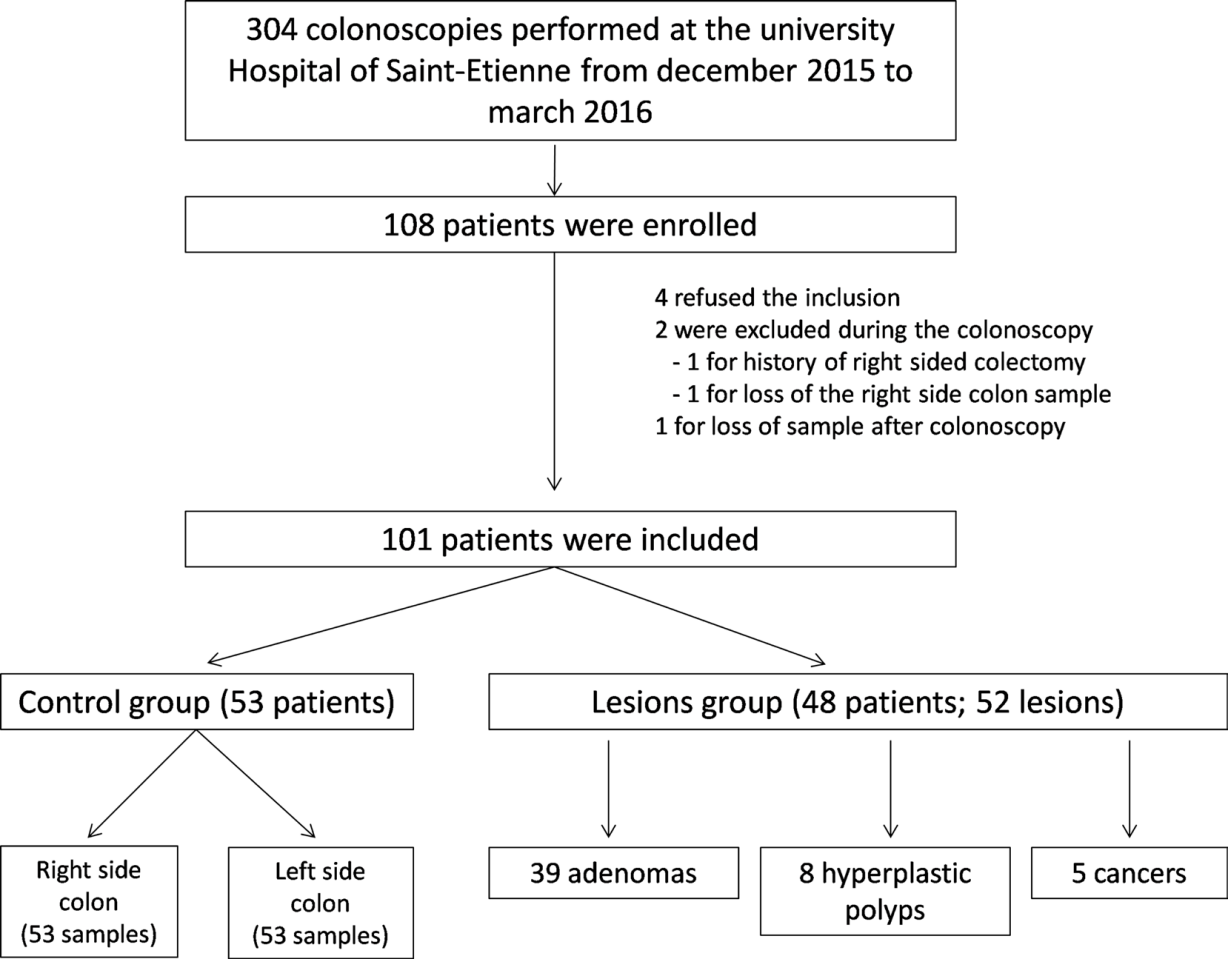
Flowchart of the population study

In total, 101 patients were included for analysis: 48 in the group with colorectal lesion(s) (Lesions group), and 53 in the group with no lesion (Control group). Of the 52 colorectal lesions biopsied, 39 (75%) were confirmed as adenomatous, 8 lesions were finally hyperplastic polyps, and 5 tumors were confirmed as lieberkuhnian adenocarcinoma (Figure 1). All demographic and endoscopic characteristics of the lesions are reported in Table 1. The 3 subgroups of lesions (adenomatous, hyperplastic, carcinomatous) were statistically distinct by their median size, superior in the cancers subgroup (35 mm, vs 8 mm and 6.5 mm, respectively; *p* = 0.03). In contrast, no statistical difference was relevant regarding lesion localization (right or transverse side colon in about 40%), their form according the Paris endoscopic classification (about 2/3 were sessile), or histological results of the distal mucosa (*p* = 0.91).

**Table 1:** Characteristics of the lesions

	Adenoma (*n* = 39)	Cancers (*n* = 5)	Hyperplastic polyps (*n* = 8)	*P* value^1^
Topography (*n*, %)				0.83
-rectum/sigmoid	5 (12.8)	1 (20)	0 (0.0)	
-left side colon /transverse colon	16 (41.0)	2 (40)	5 (62.5)	
-right side colon	18 (46.1)	2 (40)	3 (37.5)	
Size (mm)				
median, [IQR25–75]	8 [6–15]	35 [20–60]	6.5 [3–20]	0.03
mean, (± SD)	14 (± 13.4)	37.4 (± 23.9)	10.9 (± 9.2)	–
Form (Paris endoscopic classification)				0.72
-0–1p (pedunculated)	7 (18.4)	2 (40)	3 (37.5)	
-0–1s (sessile)	27 (71.0)	3 (60)	5 (62.5)	
-0–2a (slightly elevated)	3 (7.9)	0 (0)	0 (0.0)	
-0–2c (slightly depressed)	1 (2.6)	0 (0)	0 (0.0)	
Histology				–
-tubular	34 (87.2)	–	–	
-tubulo-villous	4 (10.3)	–	–	
-villous	1 (2.6)	–	–	
-low grade dysplasia	30 (76.9)	–	–	
-high grade dysplasia	9 (23.1)	–	–	
-differentiation				
Well	–	2 (40)	–	
Low	–	2 (40)	–	
NA	–	1 (20)	–	
Adjacent mucosa				
Macroscopic pattern : normal	38 (100)	5 (100)	8 (100)	–
Histology				0.079
-mild colitis	10 (25.6)	4 (80)	2 (25.0)	
-Normal	28 (71.8)	1 (20)	5 (62.5)	
-Hyperplasia	1 (2.6)	0 (0)	1 (12.5)	
Distal mucosa				
Macroscopic pattern : normal	38 (100)	5 (100)	8 (100)	–
Histology				0.91
-active	1 (2.6)	0 (0)	0 (0.0)	
-mild colitis	12 (30.8)	3 (60)	3 (37.5)	
-normal	21 (53.8)	2 (40)	4 (50.0)	
-hyperplasia	2 (5.1)	0 (0)	0 (0.0)	

^1^using Fisher’s exact test and Kruskal-Wallis test.

### Levels of Epidermal growth factor receptor expression

**Figure 2:**
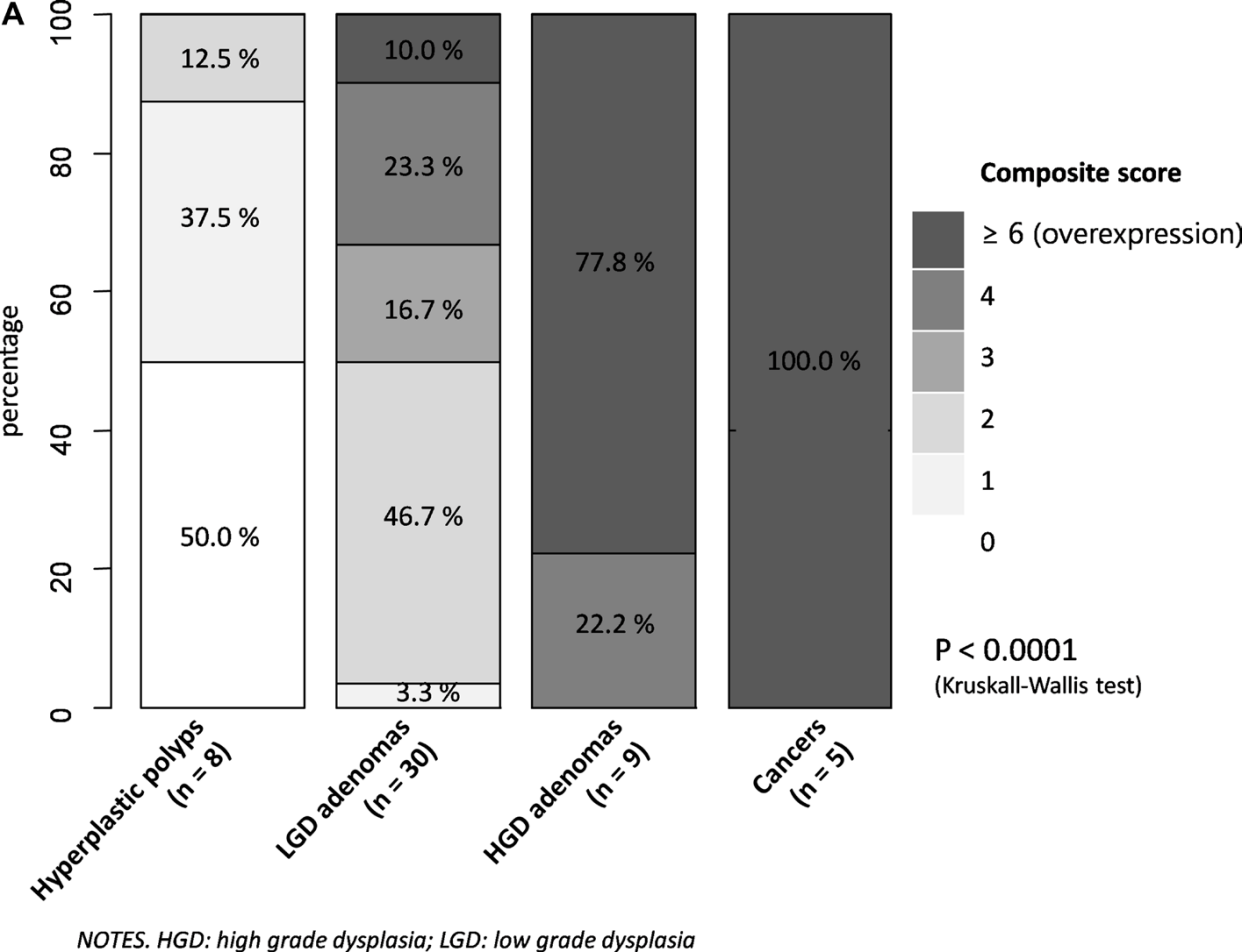

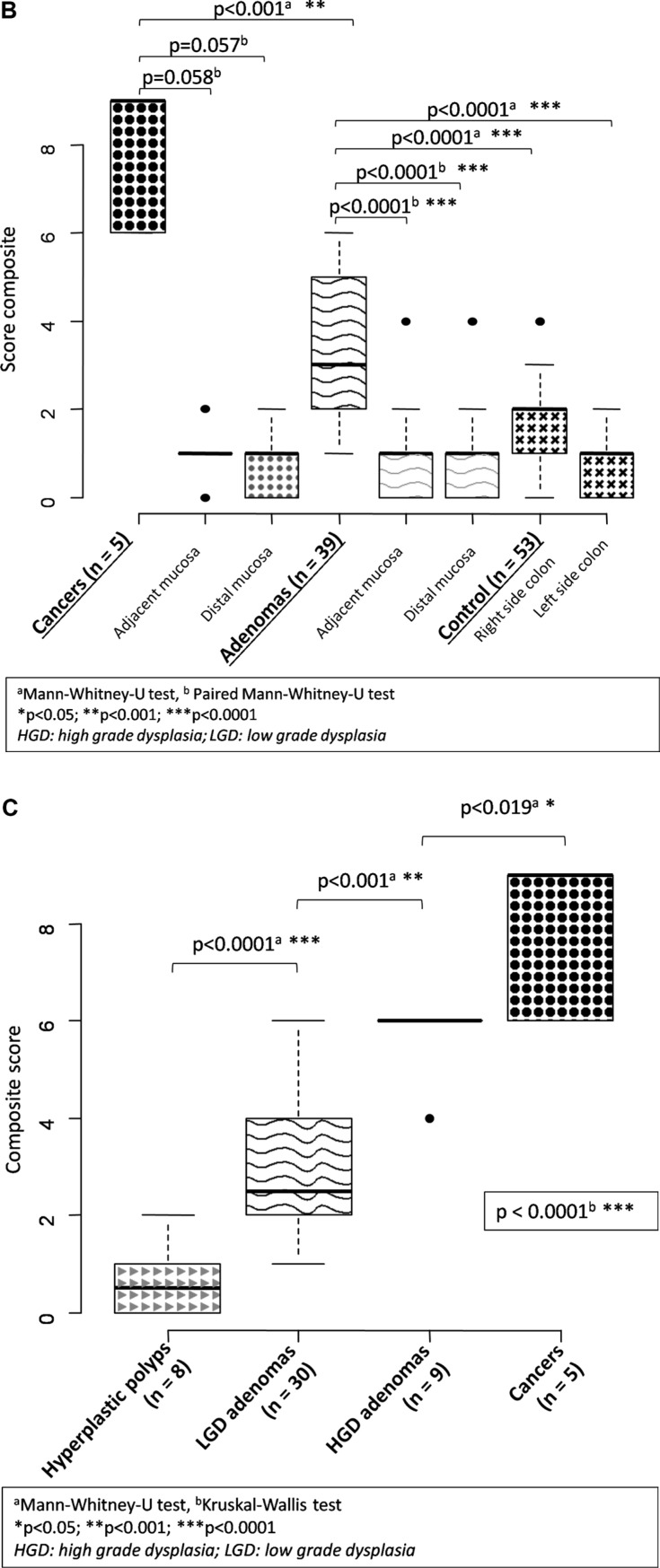
EGFR expression of the lesions assessed with the composite score (**A**) frequency per unit of composite score and type of Lesion; (**B**) Boxplots of composite scores comparing lesions and controls; (**C**) Boxplots of composite scores comparing all lesions there between.

**Figure 3:**
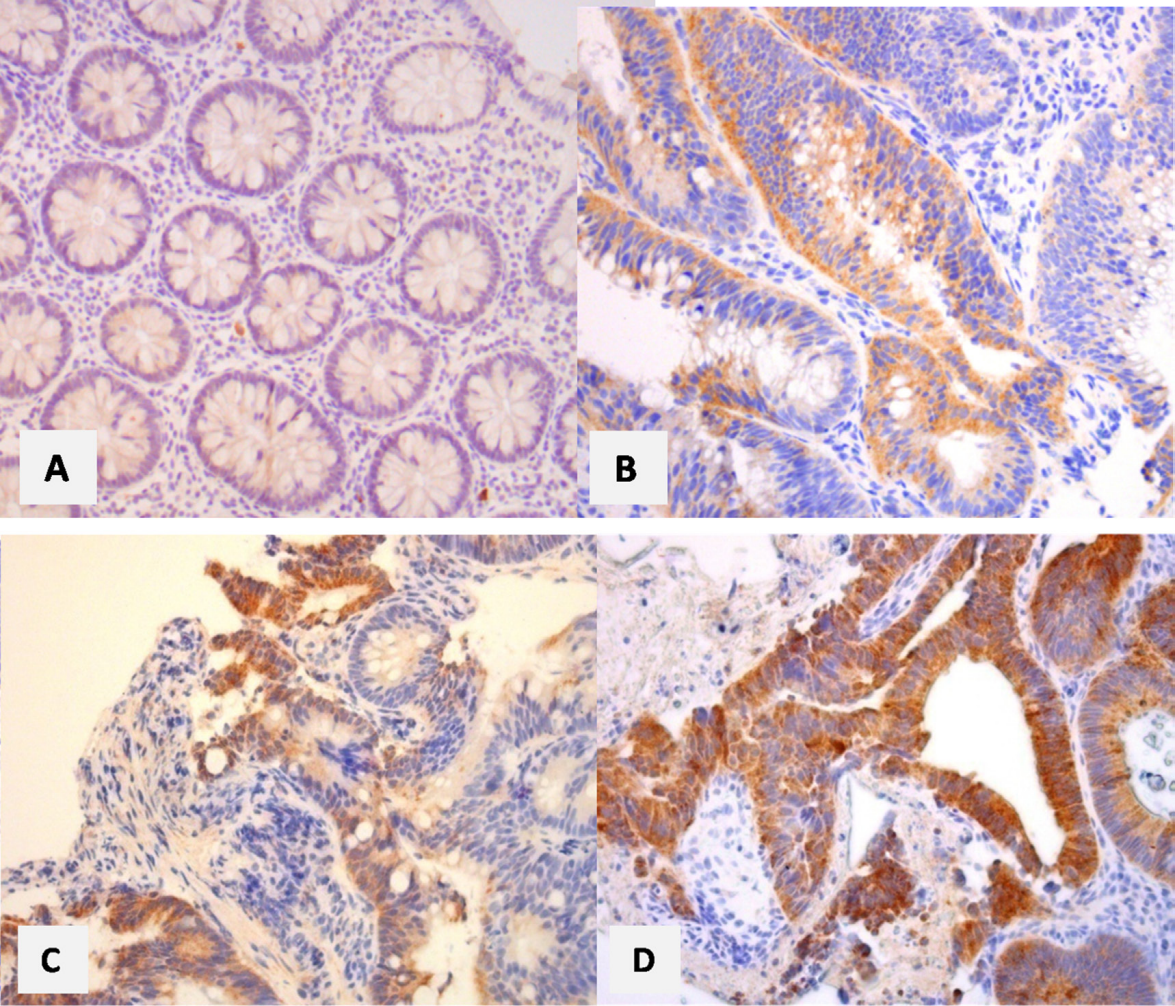
Examples of immunohistological scoring of the lesions study

Immunohistological outcomes were represented for each subgroup of lesions (adenomas, hyperplastic polyps, cancers) and for the control group, in Supplementary Figure 1 (Goldstein grade), Supplementary Figure 2 (intensity staining) and in Figure 2 (composite score). Some examples of the immunohistochemical scoring are reported in Figure 3.

All adenomatous lesions and cancers expressed EGFR with higher proportions of labeled cells per sample (Adenomas: 54.9% grade 2–3; cancers: 100% grade 3), compared to hyperplastic polyps (grade 2–3: 0%) or normal colonic mucosa issued from the control group (grade 2–3: 9.4%–28.3%) (*p <* 0.01). The median Goldstein grade was also statistically higher in cancers subgroup (grade 3; IQR_25-75%_: 3–3), compared to the adenomas subgroup (grade 2; IQR_25-75%_: 1–2) (*p* = 0.0002). Similarly, adenomas had a corresponding value that was higher than in mucosa of the right (grade 1; IQR_25-75%_: 1–2); *p <* 0.01) and left (grade 1, IQR_25-75%_: 0–1) side colon. One gradient of EGFR expression was observed within the adenomas subgroup, and specifically across the grade of dysplasia: almost the half of LGD adenomas had a Goldstein grade 2-3 vs all of those in high grade dysplasia (HGD) (*p <* 0.001). Adenomas also expressed more EGFR than their adjacent (*p <* 0.0001) or distal (*p <* 0.0001) mucosa. This difference is less marked between cancers and their adjacent (*p* = 0.054) and distal (*p* = 0.053) mucosa.

The staining intensity of EGFR was higher in subgroups of adenomas (grade 2–3: 87.1%) and cancers (grade 2–3: 100%), compared to hyperplastic polyps (max: grade 2) and control group (max: grade 2: 7.5% to 34%) (*p <* 0.0001). The intensity of EGFRs expression was similar in cancers vs adenomas (*p* = 0.66), especially for the median value of the intensity score (3 vs 2, respectively; *p* = 0.21). In contrast, adenomas expressed more intensively EGFRs than control group (median score: 1; *p <* 0.0001). Adenomas with LGD had moderate (60%) to high (23.3%) intensity of EGFR expression, which is significantly higher than hyperplastic polyps that had a null to mild EGFR expression in 87.5% (*p* = 0.0015). This difference is also observed between adenomas and their adjacent (*p* = 0.057) or distal (*p* = 0.053) mucosa.

The composite score ranged from 0 to 9, with a statistically significant difference between adenomas, cancers, hyperplastic polyps, and the control group (*p <* 0.0001). This difference is also observed by comparing exclusively adenomas with cancers (*p <* 0.001), or adenomas with control group (*p <* 0.0001). The median value of the composite score was 9 [6–9] in cancers vs 3 [2–5] in adenomas (*p <* 0.001). A difference is also observed between the median score of adenomas vs control group (*p <* 0.0001). Adenomas with HGD had an overexpression of EGFR (score ≥ 6) in 77.8% vs 10.0% in LGD adenomas (*p <* 0.0001). Half of adenomas with LGD had a moderate expression (score 3–4), which is significantly higher than hyperplastic polyps that had null or mild expression (score 2–3) in half of them. Median values of the composite score were statistically different (*p <* 0.0001) between hyperplastic polyps, LGD and HGD adenomas. This difference is particularly marked between adenomas and their adjacent (*p <* 0.0001) and distal mucosa (*p <* 0.0001), while there was a trend toward without reaching the statistical significance between cancers and their adjacent (*p* = 0.058) and distal mucosa (*p* = 0.057).

### Factors associated with overexpression of EGFR (composite score ≥ 6)

Outcomes of uni and multivariate analysis assessing factors that were associated with a composite score more than 6 were reported in Table 2. Characteristics of patients (sex, age > 50 years, tobacco status) were not associated with overexpression of EGFR. Nor the topography, nor the form of adenomas were related with the level of EGFR expression. In contrast, size of the lesion > 10 mm increase the probability of EGFR overexpression (OR: 1.45 [1.12–1.89]; *p* = 0.008). This statistical association was not confirmed by multivariate analysis that has only shown HGD (vs LGD; OR: 1.98 [1.64–2.40]; *p <* 0.0001) and tubulovillous feature (vs tubulous; OR: 2.31 [1.77–3.01]; *p <* 0.0001) as associated with a composite score ≥ 6.

**Table 2:** Uni- and multivariate analysis of factors associated with EGFR overexpression in adenomas

	*P* value Univariate analysis^1^	Odds ratio [IC 95 %]	*P* value Multivariate analysis^1^	OR [IC 95 %]
Female	0.24			
Age > 50 yrs	0.76			
Active smoker	0.85			
Topography of the lesion				
-Rectum/sigmoid	0.76			
-left side colon /transverse colon	0.42			
-right side colon	0.32			
Form (Paris endoscopic classification)				
-0–1p (pedunculated)	0.89			
-0–1s (sessile)	0.88			
-0–2a (slightly elevated)	0.88			
-0–2c (slightly depressed)	0.57			
Size ≥ 10 mm	0.008	1.45 [1.12–1.89]	0.67	
High grade vs low grade dysplasia	< 0.0001	1.97 [1.53–2.54]	< 0.0001	1.98 [1.64–2.40]
Histology				
-tubular	< 0.01	0.54 [0.37–0.77]		
-tubulo-villous	< 0.001	2.29 [1.57–3.35]	< 0.0001	2.31 [1.77–3.01]
-villous	0.56			

^1^using Logistic regression analyses.

95% CI : 95% confidence interval ; OR : odds ratio.

## DISCUSSION

### New findings

In this study, we have demonstrated that EGFRs were expressed in all adenomas, regardless the immunohistological score/grade used (proportion of labeled cells, intensity of expression, or composite score). More interestingly, the EGFR expression in lesions that were falsely considered as adenomatous by endoscopist during the colonoscopy (hyperplastic polyps) was null (50%) or mild (score 1: 37.5%; score 2: 12.5%) for all of them (*n* = 8), similarly to adjacent (*n* = 52) or distal (*n* = 52) normal colorectal mucosa (intensity score 0–1: 66.1% to 92.5%). This whole of data leads to validate EGFR as a potential biomarker of colorectal adenomas. In the large recent retrospective study of Tjalma et al, EGFR expression was moderate (40%) to strong (10%) in half of adenomas with HGD (*n* = 70). This expression was also significantly superior to adjacent normal colon crypts (*p <* 0.001), and was confirmed during an *ex vivo* colonoscopy prodecure performed in 14 mices, by the use of a cetuximab (anti-EGFR)-tracer that could clearly delineate all colorectal adenomas. With 77.8% and 100% of overexpression in HGD adenomas and in cancers respectively, the EGFR expression seems higher in our study. This could be due to the use of another primary anti EGFR antibody (clone 113) for the immunohistochemical examination in our study.

For the first time, we have also demonstrated that there is a gradient of increased EGFR expression during the colorectal carcinogenesis, from adenomas with LGD (overexpression: 10%), and HGD (77.8%), to carcinoma (100%). This finding confirms the early and growing involvement of EGFRs during the colorectal carcinogenesis. To date, this gradient had been largely reported in CRC [10, 11, 15, 22], with higher expression of EGFR in the front of tumor invasion vs the surface of the tumor, and in metastasis localizations compared to the primary tumor. Moreover, carcinomas with EGFR overexpression were associated with poor prognosis [10]. This overexpression in CRC varied from 40% to 80% across previous studies. The high sensitivity for EGFR in our 5 CRC is due to the clone 113 antibody that we chose precisely for this performance. However there are other primary antibodies that showed similar outcomes to assess the EGFR expression both in CRC and colorectal adenomas [23]. Others reported an higher incidence of EGFR expression in tubulovillous (100%) than in tubular adenomas (63% to 75%) [24], which is in concordance with our results since the tubullovillous nature was more associated with EGFR overexpression in the present study compare to tubular adenomas (OR: 2.31 [1.77–3.01]; *p <* 0.0001).

For the first time, characteristics of patients and adenomas were prospectively collected and analyzed by uni- and multivariate logistic regression to determine those that would be associated with EGFR overexpression in colorectal adenomas. Whether size > 10 mm is one well established definition criterion of advanced neoplasia, association with EGFR overexpression was not confirmed in this study by multivariate analysis (*p* = 0.67), in contrast with other co-factors such as HGD (OR: 1.98 [1.64–2.40]; *p <* 0.0001) and tubulovillous or villous feature (OR: 2.31 [1.77–3.01], *p <* 0.0001). Bansal et al. [25] showed previously that EGFR was overexpressed in large tubular adenomas >10 mm in dimension but not in smaller lesions. In contrast, in the present study there is a high rate of EGFR expression in adenomas whose size was mostly < 10 mm. This difference between these studies and others could be due to the difference of sensitivity of the primary antibody used for IHC. The primary endpoint of our study was to demonstrate the EFGR expression on colorectal adenomas, and that is the reason why we used the clone 113 anti-EGFR that had yet been reported as very sensitive for EGFR expression in CRC [10, 11]. Thereby, we show for the first time a gradient of EGFR expression whose level increase with the advancement stage of the colorectal carcinogenesis.

In contrast, status of current smokers does not impact on the level of EGFR expression. The link between Tobacco status and EGFR expression was interesting to assess since the role of tobacco in the EGFR activation had already been demonstrated in pulmonary endothelium [26]. Moreover, tobacco is known as a risk factor of colorectal polyp development [27]. Our results suggest that tobacco does not use EGFR pathway to activate polyp formation. Finally, inflammation is not a factor that was associated with EGFR overexpression.

### Strengths of the study

Strenghts of our study is the immunohistochemistry method that was very clearly detailed, such any laboratory can perform the same immunohistochemical examination. This method was based on that previously published about CRC with excellent outcomes [10, 11]. The primary objective of our study being focused on adenomas, it was necessary to use a primary antibody that was yet validated for EGFR expression in CRC. However, interobserver reproducibility was not statistically assessed, but some good examples are reported in Figure 3, and illustrate the facility for identify the level expression of EGFR, regardless the grade/score used. To recently, we had the largest series of adenomas on which EGFR expression was measured. Most of other studies focused on CRC [10–12, 14–18]. In 1990, Koretz et al., had reported outcomes in 25 colorectal adenomas, in addition to those in 114 CRC and 88 samples of normal colonic cells [12]. Only 40% of these adenomas expressed EGFR. No details were available regarding immunochemistry method. Finally, our study is the first one that collected data prospectively, leading reliability in the data collection especially for clinical and endoscopic characteristics, and for assessing potential factor that would be associated with EGFR overexpression.

### Limits of the study

Goldstein grade and so, composite score were maybe underestimated by the nature of samples. Indeed, samples issued from biopsies are known to be exposed to heterogeneous bias, as well as EGFR expression is known to show a heterogeneous staining pattern, also in adenomas. Moreover, adenomas are also heterogeneous lesions [13]. Despite CRC are also lesions with heterogeneity, there is at least adenoma with HGD, which are lesions with EGFR overexpression in 77.8% in our study. Hence, there is probably no immunonegative area on CRC surface. All CRC we reported (*n* = 5) had a maximal Goldstein grade, i-e., all surface of tumors expressed EGFRs. Another limit of our study is that we assessed no adenoma in context of Lynch syndrome (but this setting affects 5% of CRC), nor flat adenomas, such as serrated adenomas, that represent the main lesions missed during colonoscopy and are responsible of interval CRC and likely one third of CRC [28]. Moreover, different pathways are involved in colorectal carcinogenesis in Lynch syndrome vs sporadic context and IBD. All polyps resected during the period study are from the tumor suppressor pathway, while we know now that up to 35% of all adenomas, in particular those that arise from the right colon come from the alternate serrated pathway [28]. One the other hand, there was only one patient with IBD (ulcerative colitis) in the present study. This observation is not related to selection from clinicians but likely due to hazard bias, and the small sample size of the present study. Hence, a generalization of our results cannot be made regarding adenomas occurring from serrated pathway, nor for those occurring in Lynch syndrome setting or IBD. It is interesting to note that (mild) inflammation related to IBD did not increase the EGFR expression of the macroscopically normal mucosa in this patient (Goldstein grade: 1 in both side of the colon; intensity of staining and composite score ranked from 1(left side) to 2 (right side colon)). In a recent study [13], there was no difference between Lynch adenomas and sporadic adenomas in terms of EGFR expression. It would be interesting to assess these lesions with the clone 113 that we used for immunohistochemistry, despite there is probably no major difference regarding EGFR expression.

### Perspectives

The specificity of EGFR to adenomas and the gradient of their expression across the grade of dysplasia are two interesting findings for the conception of further fluorescence nanoparticle able to bind EGFR and that would be delivered during colonoscopy. By this way, all advanced colorectal adenomas should be detected, regardless its size and its form. This nanotechnology could be useful in the case of multiple polypoid lesions, such as pseudopolyp in inflammatory bowel disease, to identify this or those that are truly adenomatous and so that should be removed. Few preliminary studies had demonstrated the feasibility of such nanotechnology during colonoscopy [13].

## MATERIALS AND METHODS

### Population study

From December 2015 to March 2016, all patients who had colonoscopy whatever the indication, in the university hospital of Saint-Etienne (France), were eligible for the study. Demographic data (age, sex, Tobacco status) were collected, in addition to the clinical context related to the individual risk for colorectal adenomas (sporadic, personal or familial history of CRC, inflammatory bowel disease, Lynch syndrom). A data anonymization was performed. A signed consent was required for the inclusion of each patient. This study was approved by our local ethic committee (1849323 v 0).

Patients who did not accept to participate in study were excluded, as those who had history of colectomy, or a severe coagulation dysfunction. The lengthening endoscopic procedure time, related to the study, should not exceed few minutes.

### Nature of sample

Biopsies on the colonic mucosa were performed during colonoscopy and immediately sent to the pathology department. Patients were divided into two distinct groups: 1) patients with colonic lesion considered as adenoma (Lesions group); 2) patients with no colorectal neoplasia (Control group). In group 1, biopsies were performed on the surface of adenoma (assay N°1), its adjacent mucosa (assay N°2), and on distal colonic mucosa (assay N°3), e.g., in the opposite segment of the colon (right versus left side colon). Endoscopic characteristic of the lesion were reported by operator, such as size estimated in mm, morphology type according the Paris endoscopic classification of superficial neoplastic lesions [29] and localization in the colon (rectum, sigmoid, left side, transverse or right side of the colon). Several lesions could be biopsied in the same patient. In case of small lesion (< 10 mm), removal piece was accepted for histologic and immunohistological examination related to the study. In group 2, one biopsy was performed in the right side (assay N°1) and the left side of the colon (assay N°2). The macroscopic aspect (inflammatory or normal) of the mucosa was reported by operator in the data collection set.

### Routing and pretreatment

Samples were immediately fixed with 10% formaldehyde during 6 to 8 hours. Tissue processing prior to paraffin embedding was performed by the BOND-III automate (Leica). Finally, after embedding in paraffin manually, samples were conserved at room temperature. Paraffin sections 4 micrometers thickness were cut by microtome (Leica) and deparaffinizated in a tank at 59°C before staining.

For each patient, one slide was used for histologic examination and one for the immunohistological examination.

### Histologic examination

After Hematoxylin and Eosin staining, nature of the lesion was identified and described according recommended practices. For normal colorectal tissue, presence of inflammation, its characteristics and its intensity were collected. For adenomatous lesions, dysplasia was graded (low or high) according to the Vienna classification [30]. Colorectal carcinomas were described by reporting degree of differentiation, mucosecretion, and, if possible, limits of the tumor invasion front [30, 31].

### Immunohistological examination

There is no standard immunohistological method for the assessment of EGFR expression. Hence, basing on previous studies reporting high rate of EGFR expression in CRC (close to 100%), we used a primary mouse monoclonal antibody (EGFR, clone 113, Novocastra), and performed as similar as possible the protocol that was described in these studies [10, 15]: this primary antibody was deposited on the tissue sections for 60 min, at room temperature, followed by biotin-labeled affinity isolated goat anti-mouse immunoglobulins and streptavidin-coupled horseradish peroxidase. Complexes were visualized (brown) with 3,30-diaminobenzidine (Dako) and the slides were counterstained with Mayer’s hematoxylin, dehydrated and mounted with Peramount. Whole steps of IHC were performed by the automate BOND-III (Leica). Tonsil mucosa was used as positive control, and skeletal muscle tissue as negative control.

All slides were scored by two pathologist (CAP, MP), blinded for all patient characteristics, and using a semi quantitative grade (percentages of labeled cells), a qualitative grade (intensity of staining) and a composite score as followed: the percentages of labeled cells was graded from 0 (no positive cells), 1 (from 1 to 25% labeled tumor cells), 2 (25–50%) to 3 (> 50% positive tumor cells) according the semi quantitative Goldstein classification [32]. The intensity of staining by peroxydase deposits was scored visually from 0 (negative), 1 (weak: light beige), 2 (moderate: brown), to 3 (strong: dark brown). The composite score, previously used in main studies [10, 15], was obtained by multiplying the Goldstein grade by the intensity score. Overexpression of EGFR was defined as a composite score ≥ 6. In the case of differing opinions between the two pathologists, the definitive assessment was obtained by consensus.

### Statistic method

Quantitative variables were expressed as median with interquartile ranges (IQR) from 25% to 75%, and compared with the Mann-Whitney-U or Kruskal-wallis test, appropriately. Number and percentages of qualitative variables were reported and compared using the chi-squared test or Fisher’s exact test. All tests were performed using R, version 3.2.2. Uni– and multivariate analysis were performed to identify factors that were statistically associated with EGFR over expression. The significance level was defined as *P* ≤ 0.05.

## CONCLUSIONS

EGFRs are early expressed in all colorectal adenomas with a gradient increased expression correlated with the grade of dysplasia. The level of EGFR expression being significantly higher in adenomas compared to normal colorectal mucosa, this receptor can be validated as a surface biomarker of colorectal adenomas, and should be considered as an interesting target in the development of further nanotechnologies.

## SUPPLEMENTARY MATERIALS FIGURES AND TABLES



## Abbreviations

CRC: Colorectal cancer
EGFR: Epidermal growth factor receptor
HGD: High grade dysplasia
HP: Hyperplastic polyps
IHC: Immunohistochemistry
LGD: Low grade dysplasia
NIR: Near-infrared.
