# Supplementary material for: The level of epidermal growth factor receptors expression is correlated with the advancement of colorectal adenoma: validation of a surface biomarker
Source: Oncotarget. 2017 Feb 1;8(10):16507–17. doi: 10.18632/oncotarget.14961 (PMC5369981; doi:10.18632/oncotarget.14961)
Supplement: Supplementary file 1 [file oncotarget-08-16507-s001.pdf]

The level of epidermal growth factor receptors expression is correlated with the advancement of colorectal adenoma: validation of a surface biomarker

Supplementary Materials

A

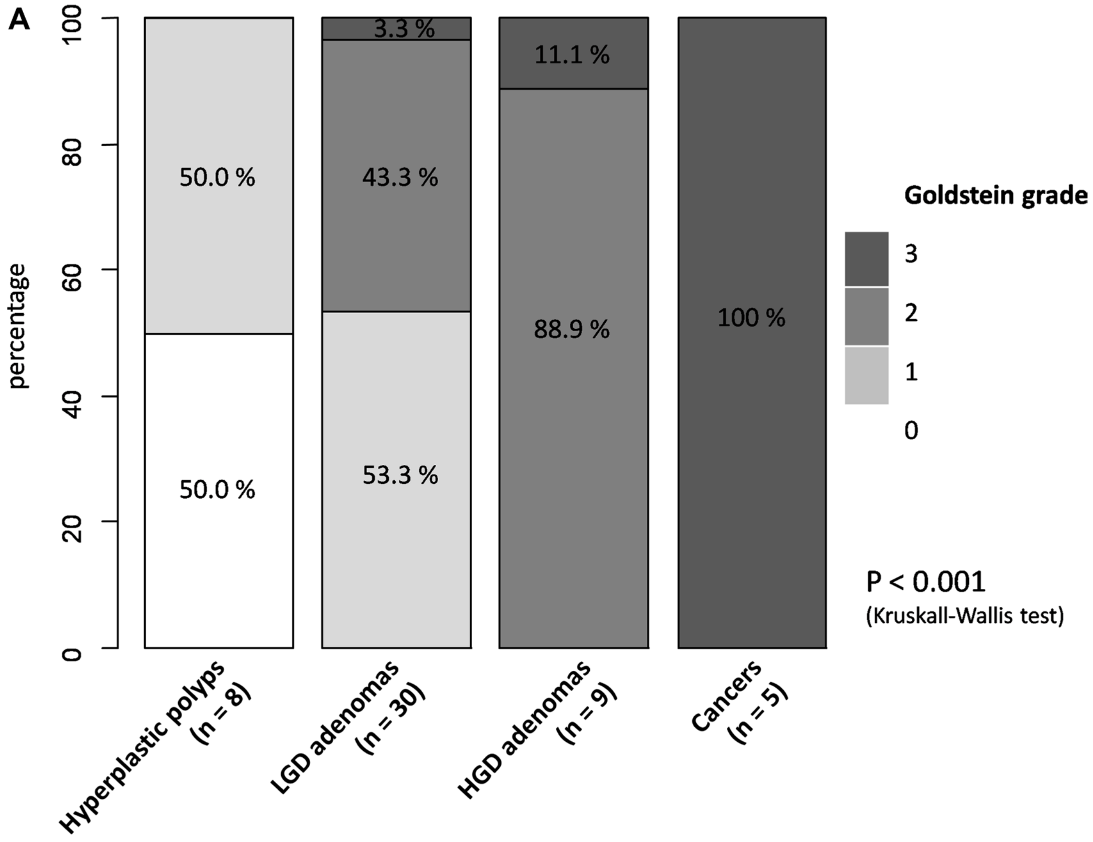

NOTES. HGD: high grade dysplasia; LGD: low grade dysplasia

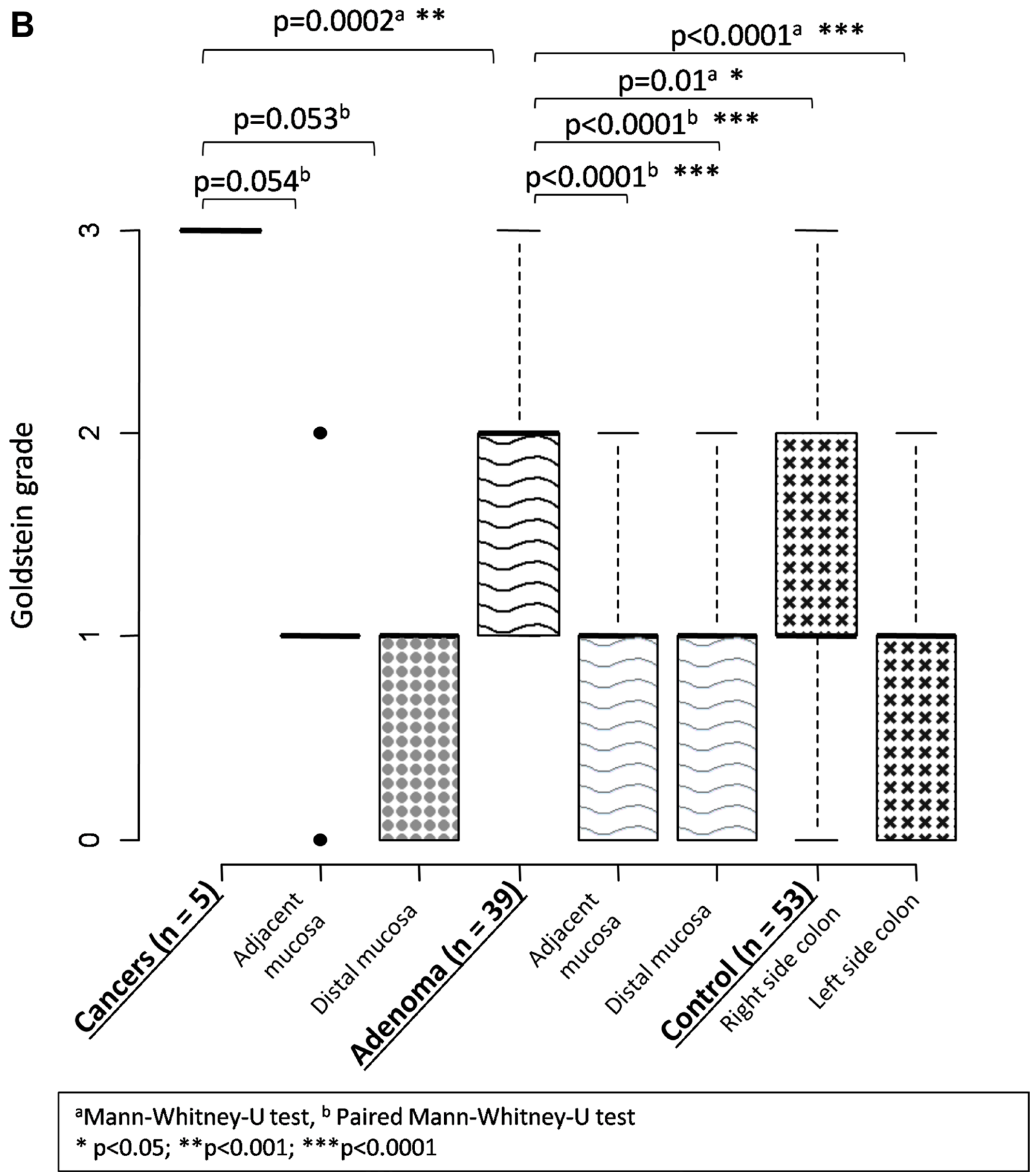

Supplementary Figure 1: EGFR expression of the lesions assessed with the Goldstein grade. (**A**) frequency per unit of Goldstein grade and type of Lesion; (**B**) Boxplots of Goldstein grade comparing lesions and controls.

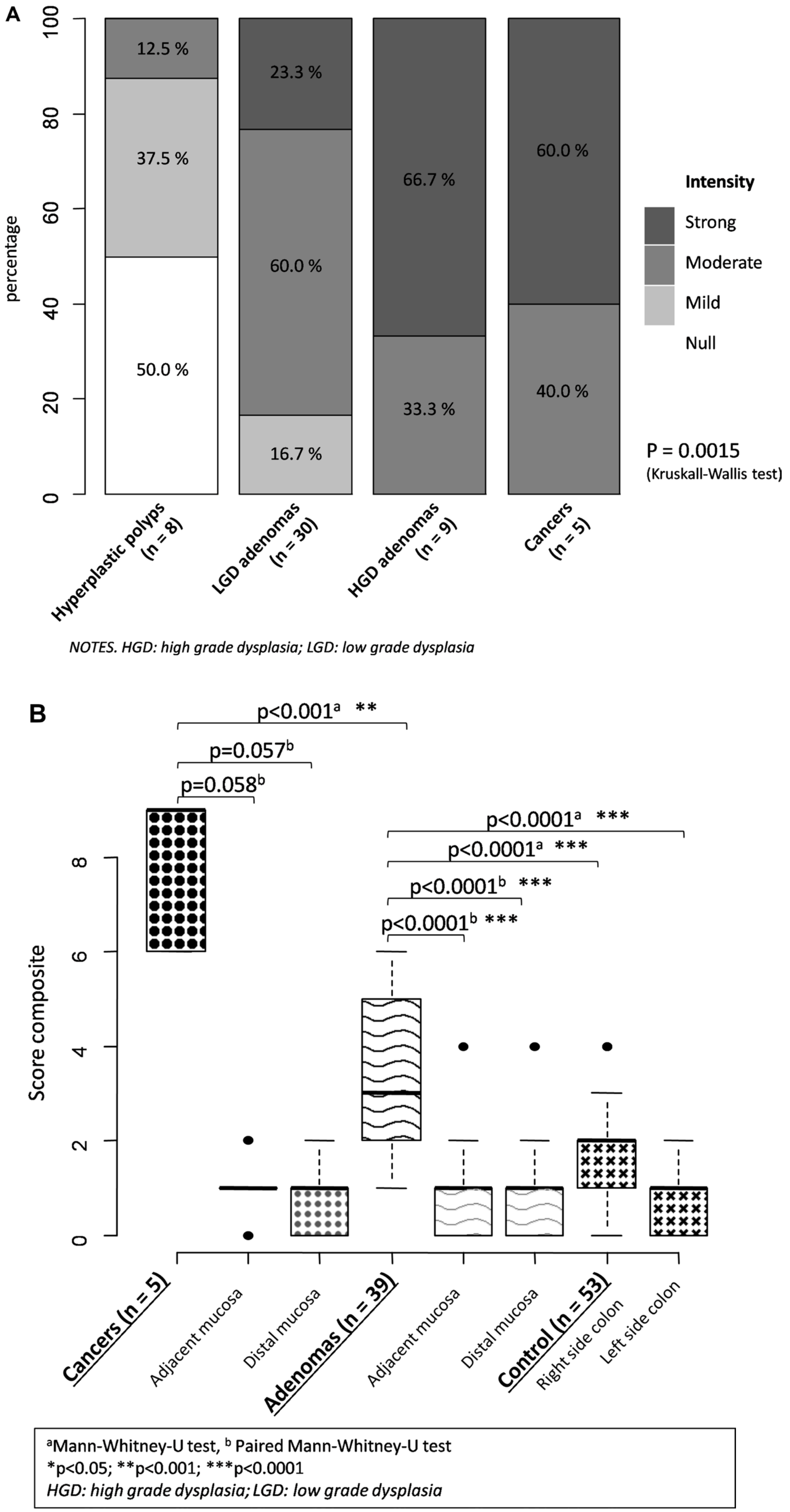

Supplementary Figure 2: EGFR expression of the lesions assessed with the intensity staining. (**A**) frequency per unit of intensity staining and type of Lesion; (**B**) Boxplots of intensity staining comparing lesions and controls.
